# GACN: Generative Adversarial Classified Network for Balancing Plant Disease Dataset and Plant Disease Recognition

**DOI:** 10.3390/s23156844

**Published:** 2023-08-01

**Authors:** Xiaotian Wang, Weiqun Cao

**Affiliations:** 1 School of Information Science and Technology, Beijing Forestry University, Beijing 100083, China; 2 Engineering Research Center for Forestry-Oriented Intelligent Information Processing of National Forestry and Grassland Administration, Beijing 100083, China

**Keywords:** deep learning, generative adversarial network, data augmentation, plant disease recognition

## Abstract

Plant diseases are a critical threat to the agricultural sector. Therefore, accurate plant disease classification is important. In recent years, some researchers have used synthetic images of GAN to enhance plant disease recognition accuracy. In this paper, we propose a generative adversarial classified network (GACN) to further improve plant disease recognition accuracy. The GACN comprises a generator, discriminator, and classifier. The proposed model can not only enhance convolutional neural network performance by generating synthetic images to balance plant disease datasets but the GACN classifier can also be directly applied to plant disease recognition tasks. Experimental results on the PlantVillage and AI Challenger 2018 datasets show that the contribution of the proposed method to improve the discriminability of the convolution neural network is greater than that of the label-conditional methods of CGAN, ACGAN, BAGAN, and MFC-GAN. The accuracy of the trained classifier for plant disease recognition is also better than that of the plant disease recognition models studied on public plant disease datasets. In addition, we conducted several experiments to observe the effects of different numbers and resolutions of synthetic images on the discriminability of convolutional neural network.

## 1. Introduction

Agriculture is one of the most important food sources for human beings. With the rapid growth of the global population, agriculture has become increasingly important. Agricultural security has an important impact on people worldwide, especially in areas where agricultural technology is underdeveloped. Plant diseases seriously hinder agricultural production and affect food quality. Accurate plant disease recognition is crucial to ensure food security, especially in less developed countries where agricultural experts are scarce. With the spread of the internet and smartphones, agricultural practitioners can take photos of plant diseases and use plant disease recognition software to correctly classify disease types. This can reduce reliance on agricultural experts and increase productivity in the agricultural sector.

With the rapid development of convolutional neural network (CNN), remarkable progress has also been made in plant disease recognition tasks. Convolutional neural networks make full use of the end-to-end learning mode and surpass machine learning methods in plant disease recognition accuracy. Brahimi et al. [1] proposed a CNN model for tomato disease classification. The tomato disease dataset contains nine diseases and 14,828 images. Fuentes et al. [2] adopted ResNet as the network backbone and proposed a local and global class annotation method to improve recognition accuracy. Ma et al. [3] designed a deep CNN model for cucumber disease image recognition. In this study, the accuracy of identifying four diseases in cucumbers was 93.4%. Bhattacharya et al. [4] used the CNN model to classify bacterial blight, blast, and brown mark diseases of rice with an accuracy of 78.44%. Huang et al. [5] proposed first separating leaves and background and then using a pretrained universal classification model to classify diseases, with an accuracy of 87.45% on the AI Challenger dataset. Sumita et al. [6] proposed a real-time recognition method based on the deep convolution neural network of corn leaf disease, which reached 88.46% accuracy. Wang et al. [7] proposed a trilinear convolute on a neural network model, which reached 84.1% accuracy. Chen et al. [8] added SE attention module on the basis of YOLOv5 to enhance recognition accuracy, and the model identified powdery mildew and anthracnose detection rates of 86.5% and 86.8%, respectively.

However, the accuracy of a deep learning model depends on the quality and quantity of the dataset. Due to insufficient numbers or unbalanced classes of some datasets, the accuracy of deep learning models is poor, so some researchers use transfer learning techniques to classify plant diseases. Fang et al. [9] proposed an instance-based transfer learning method to solve the problem of insufficient training samples of agricultural disease images. Wang et al. [10] pretrained on the PlantVillage dataset using CNN and fine-tuned their plant disease dataset. The experimental results show that combining CNN with transfer learning can improve the classification accuracy of small datasets. Zhang et al. [11] proposed using GoogLeNet to pretrain on the ImageNet dataset and then fine-tuned on 1200 cherry leaf disease datasets, achieving 99.6% accuracy. Verma et al. [12] used a pretrained ResNet18 network to fine-tune the grape leaf disease dataset to accurately identify grape disease severity. Chen et al. [13] proposed a MobileNet that added SE attention modules and increased plant disease recognition accuracy through twice-transfer learning. Vallabhajosyula et al. [14] proposed a deep ensemble neural network method to detect plant diseases and then fine-tuned pretrained models using transfer learning techniques.

The above work has made remarkable progress in plant disease recognition. However, making a plant disease image dataset requires the participation of many agricultural experts and is laborious and time-consuming. There is often a class imbalance problem in the data collection process; that is, the number of samples in some classes is significantly less than that in others. The use of class-imbalanced datasets biases the recognition model training toward the sample class which has a majority. The problems of some plant disease datasets are insufficient quantity and imbalance among classes.

Generative adversarial network (GAN) [15] has been used to synthesize images with high visual fidelity. The high-quality samples synthesized by GAN models can now be used as additional training data for tasks such as classification [16,17] and data augmentation. Data augmentation is a common technique used to synthesize more training data, which can enhance the universalization ability of the model. In image processing, data augmentation techniques usually include image flipping [18], random cropping [19], and color enhancement [20]. In image classification tasks, models trained on class-imbalanced datasets are often biased toward the majority class. This problem can be ameliorated by applying augmented dataset techniques to minority classes. Some works [21,22,23,24,25,26] use a GAN to expand the dataset or solve the problem of class-imbalanced datasets. Refs. [21,26] use CGAN to synthesize images to augment and balance datasets, but experiments in this paper prove that synthetic images of CGAN have a low level of accuracy. The methods [22,23,24,25] use nonlabel-conditional GANs to augment or balance plant disease datasets, but the disadvantage of these methods is that GANs need to be trained separately for each class. In addition, ref. [27] used transfer learning on the samples synthesized by GAN to enhance the classification accuracy of convolutional neural networks.

However, the above methods have problems with low accuracy of synthetic images and complex training processes. To solve the problem of unbalanced plant disease datasets, we propose a generative adversarial classified network (GACN) to enhance the classification accuracy of convolutional neural networks. The GACN aims to further improve the contribution of synthetic images to the discriminability of specific classification convolutional neural network. The synthetic image of the proposed method has higher accuracy, while the proposed method can generate synthetic images of any class through one-time training. The GACN consists of a generator, discriminator, and classifier. The generator is used to synthesize images. The discriminator distinguishes between the real images and the synthetic images as much as possible. The classifier is designed to correctly classify real images and synthetic images. The GACN can be directly applied to plant disease recognition tasks, or the generated synthetic images can be used to balance the dataset to improve CNN accuracy. We evaluate our method using the PlantVillage [28] and AI 2018 Challenger datasets. We compare conditional generative adversarial network (CGAN) [29], auxiliary classifier GAN (ACGAN) [30], multiple fake class GAN (MFC-GAN) [31], balancing GAN (BAGAN) [32], and ControlGAN [33] methods in the task of balancing plant disease datasets. These five methods are the existing label-conditional methods, which can output images according to the label. The difference between GACN and the above label-conditional GANs is the addition of a classifier. GACN is designed to enhance the discriminability of a specific classification CNN, so the classifier structure needs to be the same as the specific classification CNN structure. The classifier of GACN is trained on both synthetic and real images and adds a loss function for predicting the real image class to the generator, which can encourage the generator to produce more accurate synthetic images. GACN solves the problem that existing label-conditional GANs do not consider encouraging the generator to generate synthetic images with higher accuracy for specific classification CNN. The experimental results show that the GACN performance in balancing plant disease datasets is better than that of these label-conditional GANs. In addition, compared with other plant disease recognition models studied on public plant disease datasets, the classifier of the proposed method achieves higher classification accuracy.

The contributions of this paper are summarized as follows:

- A dual-purpose model GACN is proposed in this paper. The GACN is proposed to improve the accuracy of plant disease recognition tasks. It can classify plant diseases directly or generate synthetic images that can be used to balance plant disease dataset to improve CNN accuracy.
- The proposed GACN classifier is applied to the plant disease recognition task, and its accuracy exceeds that of the current methods studied on open plant disease recognition dataset.
- The synthetic image accuracy and balanced dataset performance generated by the proposed GACN model are better than those of the existing label-conditional GANs.

The remainder of this paper is organized as follows: Section 2 introduces label-conditional GANs and plant disease recognition methods based on GAN. The proposed GACN method is described in Section 3. Section 4 discusses the performance of the proposed classifier on the plant disease recognition task and the performance of GACN on balanced plant disease datasets. Section 5 describes the conclusion and what can be done in the future.

## 2. Related Work

### 2.1. Label-Conditional GANs

The GAN comprises a generator and discriminator. The purpose of the generator is to synthesize as realistic a sample for spoofing the discriminator as possible, and the purpose of the discriminator is to distinguish as much as possible between true samples and synthetic samples. The generator and discriminator are trained against each other to reach a state of equilibrium. Addressing the problem that GAN cannot synthesize samples with labels, Mirza proposed CGAN in 2014. In a standard GAN, there are no restrictions on the synthetic sample, so a sample of a given class cannot be accurately synthesized. To address this issue, CGAN conditions the generator on additional information to direct the sample generation process. The CGAN can synthesize samples of any specified class. Therefore, CGAN can be specified to synthesize samples with specific labels to balance different classes of samples in the dataset. As a variant of CGAN, ACGAN adds a loss function for correct sample classification to the discriminator, so it can synthesize higher-quality conditional samples. ControlGAN added an additional classifier but did not add a loss function to encourage the generator to synthesize more accurate images. MFC-GAN uses fake classes to ensure the accuracy of minority class generation. BAGAN models combine processes such as autoencoder training to synthesize samples. However, these methods do not consider the classification network structure when generating images, and generators are also not encouraged to produce more accurate synthetic samples, so the synthetic samples cannot further enhance the performance of the classification models.

### 2.2. Plant Disease Recognition Model Based on GAN

Jordan et al. [21] proposed a method to enhance fruit quality classification accuracy using CGAN. The experiment showed that an accuracy of 88.75% was obtained by using synthetic image enhancement training. Zhou et al. [22] proposed a GAN-based method for grape leaf spot recognition. The study generated 1000 local spot images per class and achieved 96.27% accuracy on ResNet50 by mixing the synthetic image with the real image. Lamba et al. [23] enhanced the rice disease dataset by GAN and then used CNN for classification, achieving 98.23% accuracy. Haruna et al. [24] proposed balancing the rice leaf disease dataset with StyleGAN and achieved 93% accuracy using the fast-RCNN model. Zhao et al. [25] used DoubleGAN to form an image of unhealthy plant leaves to balance the dataset and improve plant disease recognition accuracy. Abbas et al. [27] proposed synthesizing images of tomato plant leaves using CGAN. Subsequently, they used transfer learning to train the DenseNet121 model on both synthetic and real images, further improving the accuracy of the DenseNet121 model.

## 3. Method

### 3.1. Network Structure

Nonlabel-conditional GANs are not suitable for generating images that can improve CNN accuracy because synthetic images do not have label information. However, some plant disease datasets have too few minority samples, which is not enough to support GAN training. Even the models based on label-conditional GAN, such as CGAN, ACGAN, MFC-GAN, and BAGAN, do not set the corresponding loss function to improve the accuracy of the synthetic image while generating the synthetic image. In addition, they do not consider the problem of training different CNN structures on synthetic images. In view of the above problems, a classifier is added to the GAN in this paper to enhance the accuracy of the synthetic images according to the classification results of the classifier. The classifier is trained with the generator and discriminator. The synthetic images produced by GACN can output synthetic images with higher accuracy according to specific CNN structure. The trained classifier can also be directly applied to image classification tasks.

**Figure 1:**
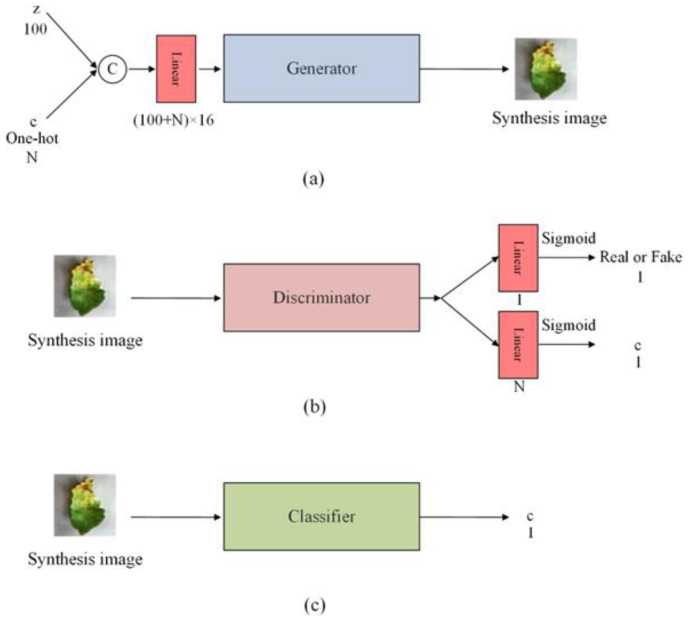
Structure of the generative adversarial classified network. (**a**) Structure of the generator. (**b**) Structure of the discriminator. (**c**) The classifier structure is the same as the specific classification network. “z” represents noise. “c” represents the label. “N” represents the number of classes.

The proposed GACN is shown in Figure 1. It comprises a generator, discriminator, and classifier. The discriminator uses convolution with a stride of 2 and finally uses nonlinear mapping layers to output a source S and a class label C. The discriminator’s structure is shown in Table 1. The structure of the proposed discriminator is similar to that of the other GAN discriminator. Most discriminators use a convolution layer with stride 2 to downsample the feature map and gradually increase the number of convolution channels. The drop operation prevents the generator’s synthetic images from being too similar due to overtraining of the discriminator. The generator structure is shown in Table 2. Each synthetic image to be synthesized $X_{fake}=Generator(c,z)$ has a corresponding class label c, which is entered into the generator as additional information in the form of one-hot encoding. The mapped latent code z and additional information are combined and fed into the generator through concatenate operations. The nonlinear mapping layer maps latent code to increase its size and reshape the feature map to $R^{C\times 4\times 4}$ before entering the generator. The generator uses a bicubic interpolation operation to upsample the feature map size before the convolution layer. Early GAN use of deconvolution as an upsampling operation produces grid-like artifacts, but bicubic interpolation operation solves this problem. Notably, the network structure of the generator and discriminator has a limited influence on the results, and the loss function is the key to generating highly accurate images. The proposed model aims to further enhance the contribution of synthetic images to specific classification network discriminability. Therefore, the classifier structure is the same as the specific classification network structure. For example, for synthetic images to enhance the ResNet performance, the classifier must have the same structure as ResNet. In this paper, we set the classifier structure to ResNet18 to compare performance with other models.

### 3.2. Objective Function

We alternately train the discriminator, generator, and classifier. The objective function of the discriminator has two parts: the log-likelihood of the correct class $L_{c}$ and the log-likelihood of the correct source $L_{s}$:

(1) $$L_{c}=E[logP(C=c|X_{real})]+E[logP(C=c|X_{fake})]$$

(2) $$L_{s}=E[logP(S=real|X_{real})]+E[logP(S=fake|X_{fake})]$$

The discriminator is trained to minimize $L_{s}+L_{c}$, where $X_{real}$ is the real image and $X_{fake}$ is the fake image. Minimizing $L_{c}$ means that the discriminator must correctly classify the classes of the real and synthetic images. Minimizing $L_{s}$ indicates that the discriminator correctly classified the true and false of the real and synthetic images. The objective function of the generator is to minimize $L_{sf}+L_{cf}+L_{tcr}$:

(3) $$L_{sf}=E[logP(S=real|X_{fake})]$$

(4) $$L_{cf}=E[logP(C=c|X_{fake})]$$

(5) $$L_{tcr}=crossentropy[Tc(X_{real}),C=c]$$

where $L_{sf}$ is the source from which the discriminator determines $X_{fake}$. $L_{cf}$ is the discriminator that determines the class of $X_{fake}$. Tc(·) is the classifier. crossentropy is the cross-entropy loss. $L_{tcr}$ calculates the distance between the classification result of $X_{real}$ by the classifier and the real label c by using cross entropy. Minimizing $L_{sf}$ indicates that the generator should make the synthetic image more realistic and cheat the discriminator. Minimizing $L_{cf}$ makes the synthetic image more consistent with its corresponding class. The objective function of the classifier is as follows:

(6) $$L_{tcf}=crossentropy\left[Tc\left(X_{fake}\right),C=c\right]\times r+crossentropy[Tc(X_{real}),C=c]$$

The classifier is trained to minimize $L_{tcf}$. r is used to control the weight of the false image to the classifier. In this paper, the value of r is 0.2. Minimizing $L_{tcf}$ indicates that the classifier correctly classified real and fake images.

In each iteration of the training process, the discriminator is trained first, then the generator is trained, and finally, the classifier is trained. In the discriminator training stage, the discriminator is trained to classify the fake and real images and determines whether the image is true or false. In the training classifier stage, the classifier is trained to correctly classify fake and real images. Additionally, we set the coefficient r, which ensures that the classifier is not more affected by poor-quality false images. In the process of training the classifier, the synthetic image classes output by the generator should be evenly distributed. For example, if the dataset contains 61 classes, with batch size set to 128, synthetic image for each class should be generated at least twice. This can solve the problem that the classifier is affected by real images of unbalanced classes. Therefore, the trained classifier can be directly applied to the plant disease recognition task. In the training stage of the generator, the generator synthesizes fake images to deceive the discriminator. In addition, in the process of training the generator, we also add the loss of the classifier predicting the true image class to the total loss of the generator to correctly classify the true image class by training on fake images. In other words, the classifier improves classification accuracy of real images by training on fake images, encouraging the generator to generate fake images with high accuracy for the classifier. Using the trained generator to produce a synthetic image can further improve the balanced dataset.

## 4. Experiment

### 4.1. Datasets and Implementation Details

We train the proposed model on the PlantVillage and AI Challenger 2018 datasets. The PlantVillage dataset is a plant disease dataset with 54,306 images in 38 classes, covering 24 types of diseases and 14 types of crops. The types of plants include grape, soybean, blueberry, cherry, orange, peach, bell pepper, potato, raspberry, squash, apple, strawberry, and tomato. The AI Challenger 2018 dataset contains 10 crops and 27 diseases, with a total of 36,379 images divided into 61 classes. Plant diseases include bacterial, mold, viral, and mite diseases. Unlike those in the PlantVillage dataset, the early crop disease images in the AI Challenger 2018 dataset are very similar to the healthy images, so they were more difficult to classify. The training and test images were set in a ratio of 8:2.

The Adam optimizer was used for the generator and the discriminator, where beta1 and beta2 were 0.5 and 0.999, respectively. The classifier uses Adam as the optimizer, where weight decay is 1 × 10^−4^. The batch size is 128. The epoch is set to 300. The learning rates of the generator, discriminator, and classifier networks were 0.0001, 0.0004, and 0.0004, respectively. All experiments were performed in Python 3.8.8, PyTorch 1.10.2, and CUDA 10.2. The Ubuntu 18.04 with NVIDIA RTX2080TI GPU was used to train and test the proposed model.

### 4.2. Evaluation Metrics

We evaluate the proposed model based on the following metrics: accuracy, PIQE [34], NIQE [35], and Inception Score [36].

(7) $$Accuracy=\frac{TN+TP}{TN+TP+FN+FP}$$

True negative (*TN*) represents the number of predicted results that are negative and actual class that are also negative. True positive (*TP*) represents the number where the predicted result is positive and the actual class is positive. False negative (*FN*) represents the number where the predicted result is negative and the actual class is positive. False positive (*FP*) represents the number of predicted results that are positive and the actual class that is negative. The accuracy represents the proportion of model prediction results to actual results. By imitating human visual behavior, the PIQE and NIQE evaluate the perceptually important areas on the image and are used to evaluate the image quality. High PIQE and NIQE scores indicate low image quality. The Inception Score is used to evaluate image quality and is commonly used to measure GAN performance. The higher the Inception Score is, the better the image quality generated by the GAN. The implementation process of PIQE, NIQE, and Inception Score can be found in the above papers.

### 4.3. Plant Disease Recognition Performance

#### 4.3.1. Comparison of Plant Disease Recognition Accuracy

We compared the trained classifier in the proposed method with several other plant disease recognition models. These plant disease recognition models are all methods studied on public datasets. The accuracy of the above models was obtained by the PlantVillage and AI Challenger 2018 test sets. As shown in Table 3, refs. [37,38,39] early plant disease recognition models used universal recognition networks to classify plant diseases, and they did not design corresponding network structures according to the plant disease characteristics. Refs. [40,41] improved the VGG model, reduced the number of parameters, and improved the performance compared with the standard VGG network [41]. The model has only 6 M parameters and, therefore, is suitable for running on mobile devices, Refs. [42,43]. By using the attention mechanism to enhance plant recognition accuracy, ref. [42] with only 0.7 M parameters, due to the effective image recognition performance of ResNet18, ref. [44] satisfactory accuracy was achieved after adding the attention model. Ref. [45] with only 4 M parameters was proposed and specifically designed for plant disease recognition tasks. The proposed model classifier has the same structure as ResNet18, and its performance is better than that of the ResNet18 variant proposed by [42]. The classifier of the proposed model is trained on both real and synthetic images, and the accuracy of the plant disease recognition task is better than that of these existing works.

To prove that GACN can improve the accuracy of the plant disease recognition task, we set up several experiments to verify the accuracy of the original ResNet and the accuracy of the GACN classifier. We compare the classifier performance with ResNet18, ResNet34, ResNet50, and ResNet101. As shown in Table 4, ResNet18 achieved 84.75% accuracy on the AI Challenger 2018 dataset. After the classifier structure in GACN is set to ResNet18, the accuracy of ResNet18 improved by 1.77% after training with real and synthetic images. When the GACN classifier is ResNet50, the accuracy is 0.49% higher than that of ResNet50 on the PlantVillage dataset. The experimental results of ResNet18, ResNet34, ResNet50, and ResNet101 show that ResNet accuracy can be improved by training real images together with synthetic images of the proposed method. The trained GACN classifier can be directly applied to plant disease recognition tasks, and the number of parameters will not increase compared with the original ResNet network.

We carried out a five-fold cross experiment to verify the accuracy of the classifier in plant disease recognition, as shown in Table 5. The five-fold cross experiment divides the dataset into five blocks, one of which is used for testing and the rest for training. When K is 1, the first block is used for testing. The maximum block accuracies in Challenger 2018 and PlantVillage are 86.52 and 99.78%, respectively. The average accuracies of AI Challenger 2018 and PlantVillage are 86.37% and 99.63%, respectively.

#### 4.3.2. Accuracy Curve during Training

**Figure 2:**
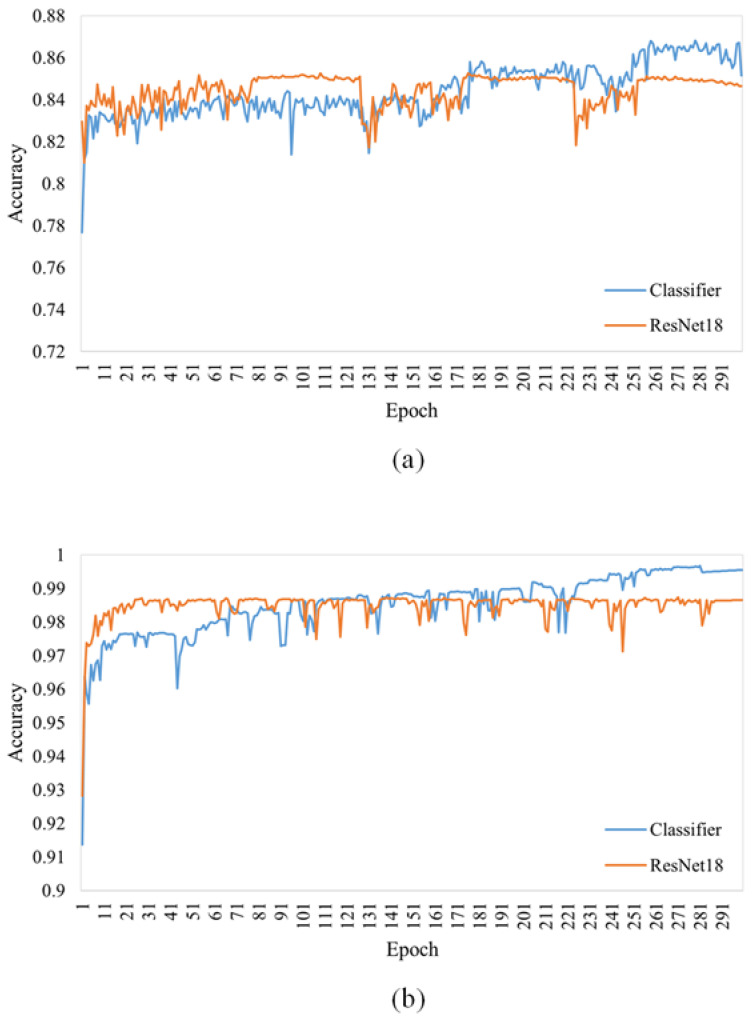
The accuracy curve of the classifier during training is compared with the original ResNet18. The classifier structure in GACN is the same as that of ResNet18. (**a**) Accuracy curve on the AI Challenger 2018 test set. (**b**) Accuracy curve on the PlantVillage test set.

Figure 2 shows the accuracy curves of the GACN classifier and the original ResNet18 on the AI Challenger 2018 and PlantVillage test sets. We can observe that the original ResNet18 is more accurate than the GACN classifier before epoch 100 on the PlantVillage dataset; however, the GACN classifier is more accurate than the original ResNet18 after epoch 150. In the AI Challenger 2018 test set, the GACN classifier was more accurate than the original ResNet18 after epoch 180. This shows that during GACN classifier training when the accuracy of the synthetic image improved, the performance of ResNet18 benefitted from the synthetic images. Therefore, the trained classifier in GACN can be directly applied to plant disease recognition tasks.

#### 4.3.3. Influence of False Image Weights on the Results during Training

**Figure 3:**
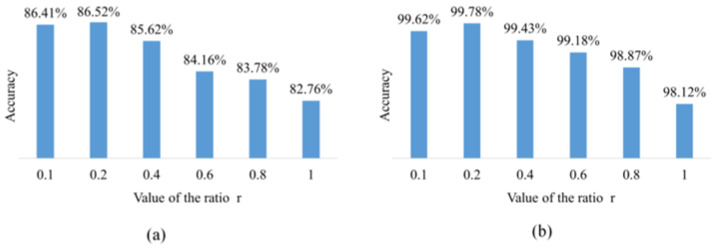
The effect of r values on results in the classifier loss function. (**a**) On the AI Challenger 2018 test set. (**b**) On the PlantVillage test set.

Figure 3 shows the effect of different ratios of r in the classifier on the results. This set of experimental results comes from a classifier trained on the AI Challenger 2018 and PlantVillage datasets. The classifier structure is the same as ResNet18. When r is 1, the synthetic and real images have the same contribution to classifier training. However, the synthetic image does not yet have enough accuracy to replace the real image, so the accuracy is lower than that of ResNet18, which is trained with only real images. As the r value decreased, it also meant that the synthetic image contributed less to classifier training and improved accuracy on the AI Challenger 2018 test set. When the value of r decreased to 0.4, the classifier accuracy is better than that of the original ResNet18 network. The experimental results show that the classifier achieves the best accuracy when the r value is set to 0.2. This proves that training with synthetic and real images can enhance the accuracy of convolutional neural networks.

### 4.4. Performance of Synthetic Images

#### 4.4.1. Performance of the Synthetic Image among Different GANs

We designed experiments to compare the accuracy of synthetic images from different GANs. Our comparison methods include only the label-conditional GANs because plant disease datasets are typically small and the number of images per class is insufficient to support nonlabel-conditional GAN method training. In Table 6, we present information from two plant disease datasets. In the PlantVillage training set, there are 152 images in the minimum minority class and 5507 images in the maximum majority class; the median is 1403, and the mean is 1150. In the AI Challenger 2018 training set, there is only 1 image in the minimum minority class and 2221 in the maximum majority class; the median is 343, and the mean is 754.

**Figure 4:**
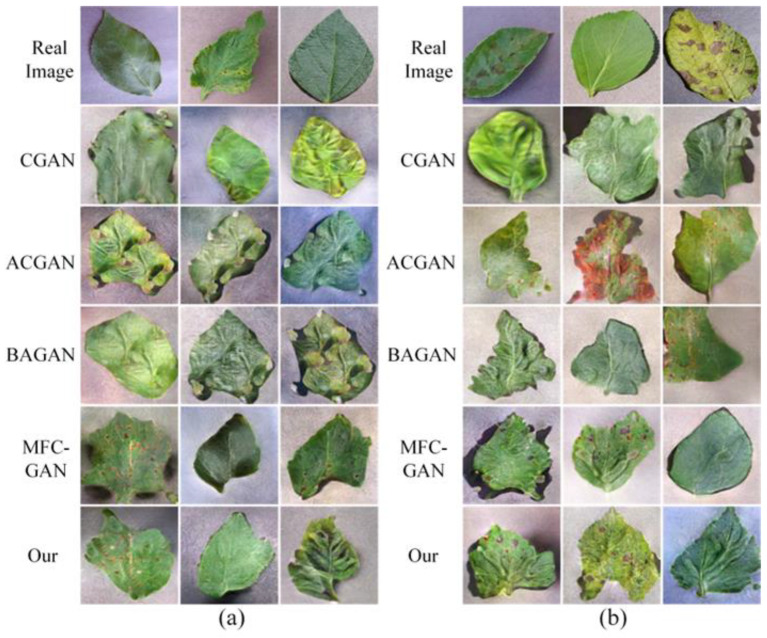
Synthetic images of different GANs. (**a**) On the PlantVillage dataset. (**b**) On the AI Challenger 2018 dataset.

In the best case, synthetic images can be used as a substitute for real images. If a GAN model can synthesize images with sufficient accuracy, it can significantly enhance the accuracy of class-imbalanced datasets. Therefore, the goal of label-conditional GANs is not only to synthesize conditional images but also to synthesize conditional images that are as accurate as possible. The synthetic images of the different GANs are shown in Figure 4.

This set of experiments was designed to verify the accuracy of synthetic images of different GANs. We use the synthetic images synthesized by different GANs as training images to train ResNet18 and verify the trained ResNet18 on the real image test set. In the set of experiments, we studied the effect of different numbers of synthetic images on the accuracy of the PlantVillage and AI Challenger 2018 test set. As shown in Table 7, regardless of how many images are synthesized by the CGAN, the accuracy of the synthetic images is always approximately 5% on the PlantVillage dataset. The ACGAN synthetic image reached its maximum accuracy of 34.9% at 36,000 images. The accuracy of the MFC-GAN synthetic images is approximately 5% higher than that of the ACGAN. The proposed model achieves the highest accuracy of synthetic images. When there were 38,000 synthetic images, the highest accuracy is achieved by training on synthetic images, and more synthetic images do not further improve the classification accuracy.

Because the AI Challenger dataset has more classes, the problem of dataset imbalance is more serious, which makes it more challenging for GANs to generate images in just a few classes. As shown in Table 8, the accuracy of the CGAN synthetic image is approximately only 2%. ACGAN had an accuracy of 23.5% in the test set when generating 1000 images per class. The accuracy of BAGAN’s synthetic images is similar to that of ACGAN. The accuracy of the synthetic image of MFC-GAN is approximately 3% higher than that of ACGAN. The proposed model achieved an accuracy of 32.4% when synthesizing 61,000 images, which is also the highest among all the compared GAN models. This set of experiments shows that synthetic images have the potential to replace real images when the accuracy of synthetic images continues to improve.

As shown in Table 9, we tested the performance of different GANs on the PIQE, NIQE, and Inception Score evaluation metrics. The average PIQE, NIQE, and Inception Score of the images in the PlantVillage dataset are given in the table. All PIQE scores of GANs are lower than those of real images, but it is evident that from a human visual perspective, synthetic images cannot be as real as real images. CGAN has the highest NIQE score, indicating its worst authenticity from a human visual perspective. The PIQE and NIQE scores of the proposed model are closest to those of real images, indicating that synthetic images from GACN are more realistic than synthetic images from other GANs. The Inception Score of GANs is higher than that of real images, indicating that the Inception Score is independent on the accuracy of the synthetic image. This set of experiments shows that the closer the PIQE and NIQE scores of the synthetic image are to the real image, the higher the accuracy of the synthetic image.

#### 4.4.2. Effect of Images Synthesized by Different GANs on Dataset Balancing

This experiment is set up so that when there are fewer images of a certain class than the specified number, the class is supplemented with synthetic images of GANs to the specified number. The experiment uses ResNet18 to train on the balanced training set and test on the real image test set to verify the performance of different GANs balanced datasets. As shown in Table 10, experiments on the PlantVillage dataset show that the CNN achieves the best performance when there are at least 1000 images per class. Replenishment of each class to 2000 images negatively affected the CNN performance. This is because the GAN is not yet able to synthesize images are accurate enough to replace the real images. MFC-GAN can improve the CNN performance by 0.4%. Balancing the training set with synthetic images of the proposed model improved the accuracy by 0.6%. However, CGAN, ControlGAN, ACGAN, and BAGAN do not improve CNN performance because their synthetic samples are not sufficient to provide sufficient discrimination ability. The experimental results show that using GAN to supplement the synthetic images in the minority class can further enhance CNN performance.

The class imbalance problem on the AI Challenger 2018 dataset is more serious. When CGAN synthetic images are added to the training set, the accuracy of the ResNet18 network decreases significantly. As shown in Table 11, synthetic images of CGAN and ControlGAN cannot yet contribute sufficient accuracy to the training set. The accuracy of the synthetic image of ACGAN is higher than that of CGAN and ControlGAN, so the performance of ACGAN is better than that of CGAN and ControlGAN in the task of balancing datasets. The synthetic image accuracy of MFC-GAN is better than that of BAGAN, ACGAN, CGAN, and ControlGAN, allowing ResNet18 to achieve maximum performance when supplementing a few classes of images up to 750 images. The accuracy of the proposed model reaches 86.3% when the class images are supplemented to 750 images. The accuracy of ACGAN, MFC-GAN, and the proposed model exceeds that of the original training set after balancing the training set. We can observe that the performance of the proposed model balance dataset is better than that of other label-condition GANs. The reason for this result is that the accuracy of synthetic images by the proposed model is better than that of other GANs, which has been demonstrated in previous experiments. This shows that when the synthetic image has a high accuracy, it can be used as an additional training image to supplement the real image dataset.

We also conducted a five-fold experiment on the task of balancing datasets, as shown in Table 12. The five-fold cross experiment divides the dataset into five blocks, one of which is used for testing and the rest for training. When K = 1, the test set is the first block. Once the dataset was divided, we used synthetic images of GACN to supplement the number of images for each class in the AI Challenger 2018 and PlantVillage training sets to 750 and 1000, respectively. We trained ResNet18 on the balanced training sets and verified its accuracy on the real image test set. The best results for the balancing dataset task were 86.3% and 99.3% in the two datasets, respectively.

#### 4.4.3. Influence of Synthetic Image Resolution on the Accuracy

As shown in Table 13 and Table 14, we study the effect of the resolution of the synthetic image on the discriminability. The experimental results show little difference in the accuracy of CGAN at 64 × 64 and 128 × 128. ResNet18 is trained on synthetic images generated by different GANs, and its accuracy is verified on the real image test set. The accuracy of the 128 × 128 samples of ACGAN is slightly higher than that of the 64 × 64 samples. BAGAN and MFC-GAN’s 64 × 64 synthetic images are approximately 2% less accurate than 128×128 synthetic images. The proposed model also achieves the best accuracy at 128 × 128 sizes. The accuracy of the 256 × 256 synthetic image is not verified by the failure of all models to generate meaningful 256 × 256 synthetic images due to the structure and loss function. This phenomenon is caused by insufficient generator parameters. Therefore, the objective of conditional image synthesis is to find the best sample resolution as far as possible to improve the classification ability of the CNN.

#### 4.4.4. Ablation Experiment

This set of experiments validated the effect of different components of GACN on the results. We trained on synthetic images generated by different variants using ResNet18 and tested it on the real images test set. The number of synthetic images for each class is 1000. As shown in Table 15, when we applied the generator and discriminator structure of ACGAN in the proposed method, the accuracy decreased by approximately 1%. When we set the classifier structure as VGG16 network structure, the accuracy of the trained ResNet18 on the real image test set decreased by approximately 9%. When the loss function $L_{c}$ of the discriminator is removed, we observe that the accuracy decreases significantly. This variant is similar to ControlGAN, which verifies the reason for the low accuracy of synthetic images by ControlGAN. The variant without classifier is similar to ACGAN and BAGAN. Therefore, the accuracy of the synthetic image by this variant is consistent with ACGAN and BAGAN. When GACN removes the classifier and loss function $L_{c}$ of the discriminator, this variant is similar to CGAN, and its accuracy is also greatly decreased. This shows that the important part of the GACN is the loss function rather than the generator and discriminator structure. Adding the loss function $L_{tcr}$ to the generator encourages the generator to generate more accurate images, where $L_{tcr}$ is Formula (5). In addition, the classifier structure should be the same as the specific classification network structure to improve the recognition accuracy of the specific classification network.

## 5. Conclusions

In this paper, we propose a generative adversarial classified network to synthesize images. The proposed GACN model consists of a generator, discriminator, and classifier. The proposed model can be identified directly for the plant disease or by generating the image of plant disease to balance the dataset. The trained classifier can be directly applied to plant disease recognition tasks, and the accuracy is better than that of existing plant disease recognition models studied on public datasets. The recognition accuracy of the trained classifier on the PlantVillage and AI Challenger 2018 datasets is 99.78% and 86.52%, respectively. To prove that the proposed method can further improve the discriminability of the classification network, we compare the proposed method with existing label-conditional GANs. The comparison results show that the proposed model is significantly superior to other label-conditional GANs in the accuracy of synthetic images. On the PlantVillage and AI Challenger 2018 datasets, the synthetic image accuracies of the proposed method are 44.2% and 32.4%, respectively. The proposed model also outperforms other comparison GANs in the task of dataset balancing. Experiments have shown that the higher the accuracy of synthetic images is, the better their performance in balancing dataset tasks. In addition, the effects of the number and resolution of the synthetic images on the discriminability of the classification network are also verified through several sets of experiments. Unfortunately, the proposed method cannot effectively generate 256 × 256 synthetic images, so it is impossible to determine whether 256 × 256 synthetic images can continue to improve the discriminability of CNNs. In future work, researchers can refer to BigGAN [46] to increase convolution channels of the generator to generate high-resolution synthetic images, which may continue improving the accuracy of synthetic images. As the accuracy of synthetic images continues to improve, the synthetic images have the potential to replace the real image training set.

## Figures and Tables

**Table 1:** Structure of discriminator. The convolution is followed by BN, LeakyReLU (slope 0.2), and dropout.

Type	Kernel	Strides	Feature Maps	Setting	Dropout	Nonlinearity
Convolution	4 × 4	2	64	-	0.5	LeakyReLU
Convolution	4 × 4	2	128	BN	0.5	LeakyReLU
Convolution	4 × 4	2	256	BN	0.5	LeakyReLU
Convolution	4 × 4	2	512	BN	0.5	LeakyReLU

**Table 2:** Structure of the generator. Feature maps are output feature map numbers. Bilinear is an upsampling mode with a scaling factor of 2 placed before the convolution. LeakyReLU (slope 0.2) is the activation function, placed after the convolution layer.

Type	Kernel	Feature Maps	Setting	Nonlinearity
Convolution	3 × 3	512	Bilinear ×2	LeakyReLU
Convolution	3 × 3	256	Bilinear ×2	LeakyReLU
Convolution	3 × 3	128	Bilinear ×2	LeakyReLU
Convolution	3 × 3	64	Bilinear ×2	LeakyReLU
Convolution	3 × 3	32	Bilinear ×2	LeakyReLU
Convolution	3 × 3	3	-	Tanh

**Table 3:** Comparison with existing plant disease recognition models on the AI Challenger 2018 and PlantVillage test sets. The classifier structure of the proposed method is the same as that of ResNet18.

Study	Year	Network	Param	AI Challenger 2018	PlantVillage
Ferentinos [37]	2018	VGG	138 M	82.71%	97.67%
Too et al. [38]	2019	DenseNet	8 M	84.56%	98.02%
Kamal et al. [39]	2019	MobileNet variant	**0.5 M**	83.78%	97.45%
Chen et al. [40]	2020	VGG19 variant	41 M	83.12%	98.74%
Ramamurthy et al. [41]	2020	CNN+attnetion	0.7 M	76.53%	96.21%
Ronghua Gao et al. [42]	2021	ResNet18 variant +attention	51 M	85.73%	99.25%
Zhao et al. [43]	2022	CNN+attention	59 M	84.23%	98.54%
Li et al. [44]	2023	CNN	4 M	85.56%	98.61%
Singh Thakur et al. [45]	2023	VGG variant	6 M	85.89%	98.75%
Our	2023	GACN (Classifier ResNet18)	11 M	**86.52%**	**99.78%**

**Table 4:** Performance comparison between the classifiers of GACN and ResNet on the AI Challenger 2018 and PlantVillage test sets.

Method	Setting	Param	AI Challenger 2018	PlantVillage
ResNet18	-	11 M	84.75%	98.74%
GACN	Classifier ResNet18	11 M	86.52%	**99.78%**
ResNet34	-	21 M	84.87%	98.81%
GACN	Classifier ResNet34	21 M	86.12%	99.26%
ResNet50	-	23 M	84.92%	98.92%
GACN	Classifier ResNet50	23 M	**86.64%**	99.41%
ResNet101	-	42 M	85.04%	99.02%
GACN	Classifier ResNet101	42 M	86.36%	99.31%

**Table 5:** Accuracy of five-fold cross experiment on the AI Challenger 2018 and PlantVillage datasets.

Datasets	k = 1	k = 2	k = 3	k = 4	k = 5	Average
AI Challenger 2018	**86.52%**	86.25%	86.46%	86.44%	86.16%	86.37%
PlantVillage	**99.78%**	99.72%	99.65%	99.43%	99.56%	99.63%

**Table 6:** The information in the dataset includes the number of classes and the distribution statistics for each class of images.

Dataset	Classes	Min	Mean	Median	Max
PlantVillage	38	152	1150	1403	5507
AI Challenger 2018	61	1	754	343	2221

**Table 7:** Accuracy comparison of the number of synthetic images among different GANs. Each class has the same number of synthetic images. The synthetic images were trained on ResNet18, and the classification accuracy was calculated on the PlantVillage test set. The synthetic image size is 128 × 128. The classifier structure of the GACN is the same as that of ResNet18.

Amount	CGAN	ControlGAN	ACGAN	BAGAN	MFC-GAN	Our
3800	5.2%	7.1%	11.1%	12.6%	15.4%	23.5%
19,000	5.8%	6.8%	33.2%	32.5%	38.2%	43.3%
38,000	5.4%	7.6%	34.9%	35.2%	39.6%	**44.2%**
76,000	5.5%	7.2%	32.1%	32.5%	39.8%	42.2%

**Table 8:** Accuracy comparison of the number of synthetic images among different GANs. Each class has the same number of synthetic images. The synthetic images were trained on ResNet18, and the classification accuracy was calculated on the AI Challenger 2018 test set. The synthetic image size is 128 × 128. The classifier structure of GACN is the same as that of ResNet18.

Amount	CGAN	ControlGAN	ACGAN	BAGAN	MFC-GAN	Our
15,250	2.3%	3.2%	9.4%	10.2%	12.5%	16.6%
30,500	2.1%	3.6%	19.2%	19.6%	20.2%	28.5%
61,000	1.9%	3.3%	23.5%	22.5%	26.1%	**32.4%**
91,500	2.2%	2.9%	22.1%	21.6%	24.7%	31.2%

**Table 9:** Average PIQE, NIQE, and Inception Score comparison of synthetic images among different GANs. The synthetic images for each class are 1000 on the PlantVillage dataset. The synthetic image size is 128 × 128. The classifier structure of GACN is the same as that of ResNet18.

Metrics	Real Images	CGAN	ControlGAN	ACGAN	BAGAN	MFC-GAN	Our
PIQE	16.42	3.47	4.67	5.51	5.34	6.87	7.62
NIQE	17.84	88.57	56.89	42.27	45.64	38.56	31.15
Inception Score	2.78	3.46	3.78	3.26	3.51	3.56	3.05

**Table 10:** Comparison results of different GANs with the balanced PlantVillage dataset. According to different datasets, the number of images for each class in the training set is supplemented by synthetic images to a specified number. The synthetic images were trained on ResNet18, and the classification accuracy was calculated in the PlantVillage test set. The synthetic image size is 128 × 128. The classifier structure of the proposed model is the same as that of ResNet18.

Amount	CGAN	ControlGAN	ACGAN	BAGAN	MFC-GAN	Our
500	96.8%	97.2%	97.3%	97.1%	97.2%	97.1%
1000	97.1%	96.7%	98.1%	98.2%	99.1%	**99.3%**
1500	96.9%	97.1%	97.2%	97.8%	98.1%	98.5%
2000	96.7%	96.6%	96.2%	97.3%	96.8%	96.9%
Real images	98.7%					

**Table 11:** Comparison results of different GAN applications with the balanced AI Challenger 2018 dataset. According to different datasets, the number of images for each class in the training set is supplemented by synthetic images to a specified number. The synthetic images were trained on ResNet18 and the classification accuracy was calculated in the AI Challenger 2018 test set. The synthetic image size is 128 × 128. The classifier structure of the proposed model is the same as that of ResNet18.

Amount	CGAN	ControlGAN	ACGAN	BAGAN	MFC-GAN	Our
500	83.2%	84.4%	84.6%	84.1%	85.5%	85.8%
750	83.9%	83.2%	85.4%	84.2%	85.8%	**86.3%**
1000	83.5%	83.6%	85.1%	84.5%	85.2%	85.1%
1500	81.8%	82.5%	83.7%	83.6%	83.3%	83.8%
Real images	84.7%					

**Table 12:** Accuracy of five-fold cross experimentation on balanced AI Challenger 2018 and PlantVillage datasets. The synthetic images were trained on ResNet18. The synthetic image size is 128 × 128. The classifier structure of the proposed model is the same as that of ResNet18.

Dataset	k = 1	k = 2	k = 3	k = 4	k = 5	Average
AI Challenger 2018	**86.3%**	85.9%	86.1%	85.8%	86.2%	86.1%
PlantVillage	**99.3%**	99.1%	98.9%	98.7%	99.2%	99%

**Table 13:** Accuracy comparison of the resolution of synthetic images among different GANs. Each class has 1000 synthetic images. The synthetic images were trained on ResNet18 and the accuracy was calculated in the PlantVillage test set.

Resolution	CGAN	ControlGAN	ACGAN	BAGAN	MFC-GAN	Our
64 × 64	5.7%	6.5%	29.7%	30.1%	38.2%	43.5%
128 × 128	5.4%	7.6%	34.9%	35.2%	39.6%	**44.2%**

**Table 14:** Accuracy comparison of the resolution of synthetic images among different GANs. Each class has 1000 synthetic images. The synthetic images were trained on ResNet18 and the accuracy was calculated in the AI Challenger 2018 test set.

Resolution	CGAN	ControlGAN	ACGAN	BAGAN	MFC-GAN	Our
64 × 64	2.3%	2.9%	21.4%	21.2%	24.9%	30.3%
128 × 128	1.9%	3.3%	23.5%	22.5%	26.1%	**32.4%**

**Table 15:** The effect of different components of the proposed model on the results. Each class has 1000 synthetic images. The synthetic images were trained on ResNet18 and the accuracy was calculated in the PlantVillage and AI Challenger 2018 test set. *L_c_* is Formula (1).

Setting	PlantVillage	AI Challenger 2018
GACN	**44.2%**	**32.4%**
ACGAN structure	43.5%	31.3%
classifier structure VGG16	35.1%	24.7%
$w/o\;L_{c}$	19.1%	11.5%
w/o classifier	35.7%	23.2%
w/o classifier and *L_c_*	5.6%	2.7%

## Data Availability

The AI Challenger 2018 and PlantVillage datasets are open-source datasets.
